# Mechanism of Ferrous Iron Binding and Oxidation by Ferritin from a Pennate Diatom*

**DOI:** 10.1074/jbc.M113.454496

**Published:** 2013-04-02

**Authors:** Stephanie Pfaffen, Raz Abdulqadir, Nick E. Le Brun, Michael E. P. Murphy

**Affiliations:** From the ‡ Department of Microbiology and Immunology, University of British Columbia, Vancouver, British Columbia V6T 1Z3, Canada and; the § Centre for Molecular and Structural Biochemistry, School of Chemistry, University of East Anglia, Norwich Research Park, Norwich NR4 7TJ, United Kingdom

**Keywords:** Ferritin, Iron, Kinetics, X-ray Crystallography, Zinc, Stopped-flow Spectroscopy

## Abstract

A novel ferritin was recently found in *Pseudo-nitzschia multiseries* (PmFTN), a marine pennate diatom that plays a major role in global primary production and carbon sequestration into the deep ocean. Crystals of recombinant PmFTN were soaked in iron and zinc solutions, and the structures were solved to 1.65–2.2-Å resolution. Three distinct iron binding sites were identified as determined from anomalous dispersion data from aerobically grown ferrous soaked crystals. Sites A and B comprise the conserved ferroxidase active site, and site C forms a pathway leading toward the central cavity where iron storage occurs. In contrast, crystal structures derived from anaerobically grown and ferrous soaked crystals revealed only one ferrous iron in the active site occupying site A. In the presence of dioxygen, zinc is observed bound to all three sites. Iron oxidation experiments using stopped-flow absorbance spectroscopy revealed an extremely rapid phase corresponding to Fe(II) oxidation at the ferroxidase site, which is saturated after adding 48 ferrous iron to apo-PmFTN (two ferrous iron per subunit), and a much slower phase due to iron core formation. These results suggest an ordered stepwise binding of ferrous iron and dioxygen to the ferroxidase site in preparation for catalysis and a partial mobilization of iron from the site following oxidation.

## Teaser

**Background:** Ferritin stores iron by ferroxidation to form a mineral core, enabling diatom blooms upon iron input.

**Results:** Ferrous iron binds solely to ferroxidase site A anaerobically. Ferroxidation kinetics has two observed phases.

**Conclusion:** Ferrous iron and dioxygen binding to the di-iron ferroxidase site is stepwise.

**Significance:** Iron storage by ferritins requires a coordinated binding of iron and dioxygen.

## Introduction

Ferritin is a ubiquitous, iron storage, and detoxifying protein found in mammals, plants, and many microorganisms. Ferritin is a 24-mer that forms a hollow sphere, takes up soluble ferrous iron, oxidizes it at di-iron ferroxidase centers, and stores the iron oxide mineral in its central cavity. Iron is subsequently released upon demand of the organism's metabolism (1, 2). Mammalian ferritins are heteropolymers of two homologous monomers that can store thousands of iron atoms in the central cavity. The heavy (H)^2^ chain contains the ferroxidase site, whereas the light chain promotes iron core formation (3). In contrast, bacterial ferritins are homopolymers in which the monomer contains both the ferroxidase site and the mineral nucleation sites. The homologous bacterioferritins additionally contain a heme group between two monomers that functions principally in iron release (4). Storage of iron is the main function of ferritins, and detoxification of iron and reactive oxygen species is a secondary protective function utilized under extreme oxidative stress (5). However, whether these roles constitute the primary function of bacterioferritins remains to be proven (1, 2).

Recently, ferritin homologs were identified in five species of pennate diatoms but not in other stramenopiles using a PCR approach (6). A phylogenetic analysis of diatom ferritin sequences with those from both prokaryotes and eukaryotes showed that diatom ferritins are clearly distinct from other eukaryotic ferritins but may be weakly associated with prokaryotic ferritins (6). Diatoms are unicellular photosynthetic organisms that play a major role in global primary production and carbon sequestration into the deep ocean (7). In many offshore areas of the open ocean, primary productivity and therefore CO_2_ uptake from the atmosphere is limited due to iron availability. These regions are sporadically pulsed with new iron inputs from dust or upwelling deep waters. Pennate diatoms readily bloom upon such iron additions and continue to grow and divide after iron levels return to a low and ambient level (8). The expression of ferritin is thought to facilitate the blooming of pennate diatoms after iron fertilization in the open ocean.

A crystal structure of recombinant, iron-soaked ferritin derived from the pennate diatom *Pseudo-nitzschia multiseries* (PmFTN) was resolved at 1.95-Å resolution (6). The structure confirmed the characteristic ferritin ferroxidase center, monomeric fold, and spherical assembly. Nevertheless, the ferroxidase center found in PmFTN shows key differences from those of other ferritins of known structure. Typical eukaryotic H chain ferritins have a di-iron ferroxidase center (9); however, three iron atoms are observed in and around the PmFTN ferroxidase center: one is found in ferroxidase site B, and the other two are positioned toward the core. An unexpected finding was that the ferroxidase site A is occupied by a water molecule. The iron atom found at site B is coordinated by three glutamate residues (Glu-48, Glu-94, and Glu-130) conserved in all ferritins. A unique site C is found in PmFTN at which iron is coordinated by only one glutamate residue (Glu-44). A glutamate is found at position 44 only in diatoms and cyanobacteria, and moreover, no third iron site is found in human H chain ferritin or other eukaryotic ferritins.

To get a better understanding of the ferroxidase reaction and iron binding in PmFTN, we have determined the x-ray structures of several PmFTN crystals soaked for various durations in ferrous iron and zinc sulfate under aerobic and anaerobic conditions. Furthermore, stopped-flow kinetic analysis was applied to determine reaction phases of the ferroxidase reaction and to understand the iron oxidation mechanism in PmFTN.

## EXPERIMENTAL PROCEDURES

### 

#### 

##### Protein Expression and Purification

The construct used for protein expression was a pET28a(+) vector containing the coding region of PmFTN genomic DNA lacking the signal peptide and plastid-targeting sequences (6). The expressed protein is missing the proline at the N terminus compared with the sequence found at UniProt entry B6DMH6. *Escherichia coli* BL21(DE3) cells transformed with the expression vector were inoculated into 2× YT medium (16 g/liter Bacto Tryptone, 10 g/liter Bacto Yeast Extract, and 5 g/liter NaCl) and grown at 37 °C to an optical density of ∼0.8 at 600 nm. Protein expression was induced with addition of 0.2 mm isopropyl β-d-thiogalactosidase. The cells were incubated at 25 °C overnight and afterward pelleted by centrifugation. The pellet was resuspended in 20 mm Tris-HCl, pH 8, 1 mm TCEP, and 5% glycerol (v/v), and the cells were lysed using an EmulsiFlex-C5 homogenizer (Avestin). Insoluble cell debris was removed by centrifugation. The supernatant was treated with DNase I type 2 and filtered through a 0.8-μm syringe filter.

PmFTN was purified using a heat shock method as described by Marchetti *et al.* (6). Briefly, the cell extract was aliquoted in 1-ml fractions, heat-shocked for 5 min at 60 °C, and put on ice for 4–5 min. The precipitated *E. coli* proteins were removed by centrifugation, and the remaining supernatant was filtered through a 0.22-μm syringe filter. PmFTN was further purified using Source 15Q (GE Healthcare) resin. The buffer used for the Source 15Q purification was 20 mm Tris, pH 8 and 5% glycerol (v/v), and the salt gradient used was 0–50% 1 m NaCl. Purified PmFTN was dialyzed into 3% sodium dithionite (w/v), 1 m sodium acetate, pH 4.8, and 1 mm TCEP to remove bound iron to yield the apoprotein. Apo-PmFTN was further dialyzed into 50 mm MES, pH 6.5, 100 mm NaCl, and 1 mm TCEP (Buffer A). The cysteine residues were alkylated by first incubating PmFTN in Buffer A supplemented with 2 mm TCEP for 2 h at 37 °C with shaking followed by the addition of 10 mm iodoacetamide and incubation for 45 min at 37 °C with shaking.

Some early preparations retained minor DNA contamination, which did not prevent crystallization but prevented accurate determination of kinetic parameters. Thus, subsequent preparations used an alternative DNA precipitation by the addition of 10 μl of 10% polyethyleneimine (w/v) (10)/ml of supernatant instead of DNase I treatment. The reaction was gently shaken for 10 min on ice, and afterward, the DNA was pelleted by centrifugation. Also, 0.5 mm EDTA was included in the buffer used for Source 15Q chromatography.

##### PmFTN Crystallization and Structure Solution

Diffraction quality crystals of PmFTN grew by vapor diffusion in a 1:1 mixture of 20 mg/ml protein in Buffer A supplemented with 2 mm TCEP and 10 mm iodoacetamide with reservoir solutions of 0.1 m sodium acetate, pH 5.5, 1.1–1.4 m ammonium sulfate, and 0.9–1.4 m sodium chloride. The crystals were soaked in mother liquor supplemented with freshly prepared 2 mm ammonium ferrous sulfate hexahydrate for a time period of 5 min, 45 min, 4 h, or overnight or with 2 mm zinc sulfate for 1 h. The crystals were transferred to a cryoprotectant consisting of mother liquor supplemented with 30% glycerol (v/v) before flash freezing in liquid nitrogen.

To obtain crystal structures from anaerobic crystals, a PmFTN protein solution was brought into a glove box (Belle Technology UK) containing a dinitrogen atmosphere maintained at less than 35 ppm dioxygen. Anaerobic diffraction quality crystals of PmFTN grew in 0.1 m sodium acetate, pH 5.5, 1.3–1.5 m ammonium sulfate, and 0.9–1 m NaCl. The crystals were soaked anaerobically in mother liquor supplemented with 2 mm ammonium ferrous sulfate hexahydrate for time periods of 75 min and 2 h. Thereafter, crystals were transferred into mother liquor supplemented with 30% glycerol (v/v) and immersed anaerobically in liquid nitrogen through a specialized port in the glove box.

PmFTN data sets were collected at the Stanford Synchrotron Radiation Lightsource on beamline 7.1 at 1-Å wavelength. Data were processed using HKL2000 to resolutions of 1.65–2.2 Å. Phases were determined using MOLREP (11) with a previously determined PmFTN crystal structure (Protein Data Bank code 3E6S) as the search model after removal of the iron and solvent atoms (6). The initial model was edited in Coot (12) and refined with Refmac5 (13). Waters were added by running Coot Find Waters in Refmac5. Anomalous dispersion data were used to identify metal sites. Anomalous maps were obtained with the program fft using the model phases. Final occupancies for metal sites were set such that the B-factors were similar to that of the coordinating residues. Data collection and refinement statistics are shown in Table 1. Figures were generated with PyMOL.

##### Ferroxidase Kinetics

Rapid kinetic experiments were carried out using a stopped-flow instrument (Applied Photophysics DX17MV). Changes in absorption on addition of ferrous iron to aerobic apo-PmFTN were measured. A 50 mm ferrous iron stock solution was prepared by dissolving ferrous ammonium sulfate in deoxygenated water, which was bubbled with argon gas for 2 h prior to use. The stock solution was acidified with 37% HCl. 10–400 μm ferrous iron working solutions were freshly prepared prior to each experimental run using the 50 mm stock solution. 1 μm apo-PmFTN in 100 mm MES, pH 6.5 and 200 mm NaCl was mixed (1:1) by the stopped-flow instrument with the various ferrous iron working solutions, resulting in a protein concentration of 0.5 μm during data acquisition. All stopped-flow experiments were carried out at 25 °C. Resulting data were fit to a single exponential function using the program Origin (v8, OriginLab). Regeneration experiments were carried out using 1 μm PmFTN containing 48 iron ions added under aerobic conditions for a fixed period (30 min or 20 h) prior to the stopped-flow experiment.

## RESULTS

### 

#### 

##### PmFTN Overall Fold

**FIGURE 1.**
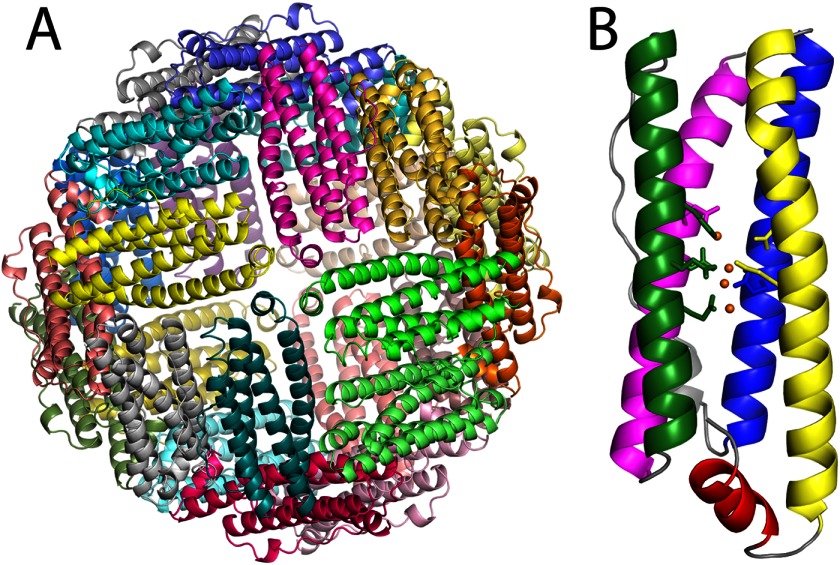
**Crystal structure of PmFTN.** *A*, crystal structure of the recombinant iron-soaked *P. multiseries* ferritin multimer shows the typical ferritin arrangement in which the 24 subunits form a spherical shell. *B*, monomer showing the ferroxidase site side chains and bound iron atoms. *Magenta*, A helix; *green*, B helix; *blue*, C helix; *yellow*, D helix; *red*, E helix; *orange spheres*, iron atoms.

Protein crystals of recombinant PmFTN soaked in Fe(II) or Zn(II) under various conditions diffracted to at least 2.2-Å resolution. Analysis of Ramachandran plots showed that in all structures more than 96% of the residues were in the preferred regions. All but one of the crystals analyzed were of space group *P*23 with eight ferritin monomers in the asymmetric unit (Table 1). The refined structures are nearly complete with at most 12 residues absent from the N or C terminus. As shown previously, the structures confirm the typical ferritin arrangement (Fig. 1): 24 subunits assemble to form a hollow spherical shell (6). The monomers adopt a four-helix bundle plus a shorter C-terminal α-helix. No significant changes in the fold of the ferritin monomer were observed upon metal treatment. Superposition of the monomers from each structure resulted in a 0.28-Å root mean square deviation for all Cα atoms.

**TABLE 1:** **Data collection and refinement statistics** Values in parentheses for the data collection statistics are for the highest resolution shell indicated. —, not applicable. ASU, asymmetric unit; o.n., overnight; ESU, estimated standard uncertainty; r.m.s.d., root mean square deviation.

	Anaerobic Fe (75)	Anaerobic Fe (2 h)	Fe (5)	Fe (45)	Fe (4 h)	Fe (o.n.)	Zn (1 h)
**Data collection**							
Resolution range (Å)	48.52–2.00 (2.00–2.05)	48.4–2.10 (2.10–2.15)	42.55–2.10 (2.10–2.16)	41.36–1.95 (1.95–2.00)	42.59–2.20 (2.20–2.26)	47.95–1.65 (1.65–1.69)	42.51–2.00 (2.00–2.05)
Space group	*P*23	*P*23	*P*23	*P*23	*P*23	*P*42_1_2	*P*23
Unit cell dimensions (Å)	*a* = *b* = *c* = 174.77	*a* = *b* = *c* = 174.32	*a* = *b* = *c* = 175.28	*a* = *b* = *c* = 175.32	*a* = *b* = *c* = 175.45	*a* = *b* = 126.25 *c* = 170.30	*a* = *b* = *c* = 175.11
No. subunits in ASU	8	8	8	8	8	6	8
Unique reflections	119,135	102,117	103,862	128,778	90,434	163,306	117,223
Completeness (%)	99.8	99.5	99.6	99.2	99.7	99.2	97.5
Redundancy	14.3	14.6	13.0	11.6	14.0	10.7	14.7
Average *I*/σ*I*	23.8 (4.8)	32.3 (5.4)	35.5 (7.1)	33.4 (3.8)	21.9 (4.7)	32.1 (3.7)	29.5 (7.4)
*R*_merge_	0.115 (0.658)	0.084 (0.597)	0.075 (0.371)	0.064 (0.528)	0.122 (0.553)	0.065 (0.507)	0.086 (0.452)
Wilson B (Å^2^)	29.8	30.3	24.2	24.8	26.2	22.4	26.9

**Refinement**							
*R*_work_ (*R*_free_)	0.187 (0.235)	0.201 (0.247)	0.176 (0.225)	0.173 (0.213)	0.187 (0.242)	0.176 (0.205)	0.184 (0.232)
Average B (Å^2^)	31.5	32.9	27.9	28.5	28.3	25.5	30.1
No. water	853	524	821	1,006	538	872	912
No. iron	8	8	6	16	35	24	—
No. zinc	—	—	—	—	—	—	32
r.m.s.d. bond length (Å)	0.020	0.019	0.020	0.024	0.019	0.028	0.021
ESU (maximum likelihood; Å)	0.099	0.116	0.102	0.081	0.120	0.051	0.096

**Ramachandran plot (%)**							
In most favorable	96.6	96.0	96.5	96.3	96.9	96.7	96.0
In allowed	3.4	4.0	3.6	3.7	3.1	3.3	4.0

**Protein Data Bank code**	4ITW	4IXK	4ITT	4IWJ	4ISP	4IWK	4ISM

##### Iron Binding to PmFTN in the Absence of Dioxygen

**FIGURE 2.**
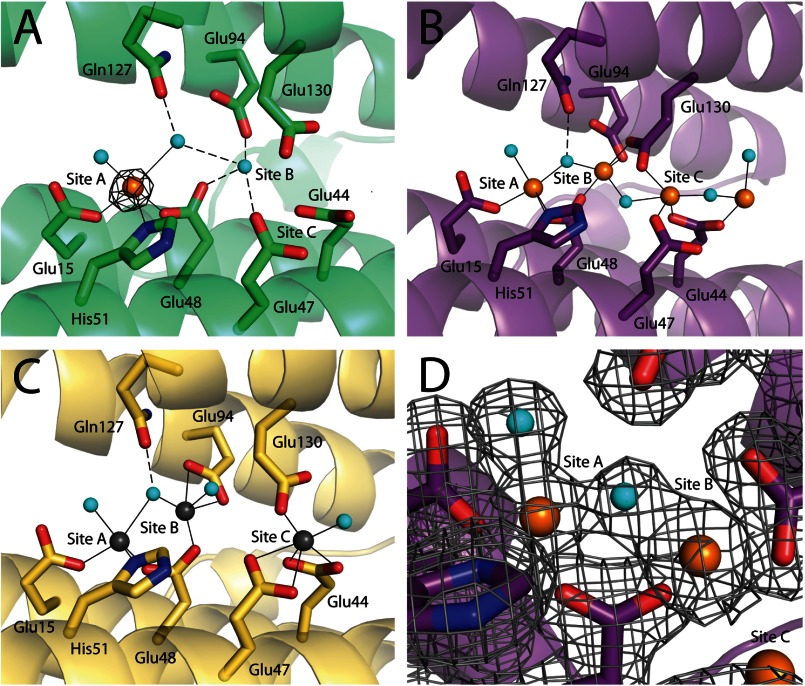
**Ferroxidase centers of PmFTN.** Monomer A of the asymmetric unit is shown. Iron and zinc atoms are drawn as *orange* and *gray spheres*, respectively, and *cyan spheres* represent water molecules. Side chains of selected residues are drawn in *sticks* with carbon, nitrogen, and oxygen atoms in the *backbone color*, *blue*, and *red*, respectively. *Solid lines* are metal ligand bonds, and *dashed lines* are selected hydrogen bonds. *A*, crystal soaked for 75 min in ferrous iron under anaerobic conditions. One iron atom is bound at ferroxidase site A at 50% occupancy, and site B is occupied by a water molecule. The *black mesh* represents the anomalous map contoured at 5 σ. *B*, crystal soaked overnight in ferrous sulfate under aerobic conditions. Three iron atoms are bound at sites A, B, and C, and one further iron atom was found beyond site C. *C*, crystal soaked for 1 h in zinc sulfate. Three zinc atoms are bound at the ferroxidase sites A, B, and C. *D*, an omit map contoured at 1 σ around site A and site B of PmFTN soaked overnight in ferrous sulfate is shown as a *gray mesh*.

The first step in the ferritin reaction is binding of ferrous iron to the ferroxidase site. To observe the binding of ferrous iron, apo-PmFTN crystals were grown in an anaerobic environment, and the octahedral crystals were soaked in 2 mm ferrous iron. Two crystal structures were obtained with crystals soaked in ferrous iron for 75 min and 2 h to resolutions of 2 and 2.1 Å, respectively. The ferroxidase site of the higher resolution structure obtained from the crystal soaked for 75 min is shown in Fig. 2*A*. Ferrous iron ions are exclusively found in the ferroxidase sites of each monomer. Furthermore, ferrous iron is observed solely in site A (Fig. 2*A*) and is refined with an occupancy of ∼50% and B-factors similar to the coordinating residues. The iron in site A is coordinated in an approximate tetrahedral arrangement by the side chains of Glu-15 (∼2.2 Å) and Glu-48 (∼2.2 Å). His-51 forms a weaker interaction with ferrous iron (2.5–2.9 Å). The coordination sphere is completed by one or two solvent molecules (∼2.1 and ∼2.5 Å) depending on the monomer in the asymmetric unit. Ferroxidase site B is occupied by a solvent molecule.

##### Iron Binding to PmFTN in the Presence of Dioxygen

**FIGURE 3.**
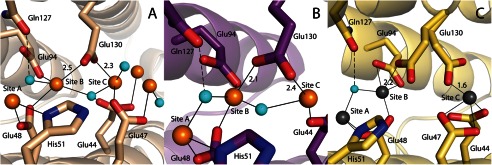
**Residue Glu-130 exhibits flexible coordination to site B and site C iron.** *A*, monomer A of PmFTN soaked in iron for 4 h. Glu-130 is coordinating to site C and may also interact with the site B iron atom. *B*, monomer B of PmFTN soaked in iron overnight. Glu-130 is coordinating to site B and may also interact with the site C iron atom. *C*, monomer C of PmFTN soaked in zinc for 1 h. Glu-130 is coordinating to the site B iron or site C iron atom. Molecular representations and the color scheme are the same as in Fig. 2. Bond lengths are in Å.

In our previous work, apo-PmFTN crystals were soaked for 10 min in ferrous sulfate in the presence of dioxygen (6). The resulting structure revealed iron bound at two sites: ferroxidase site B and a new site C nearby. Site A was occupied by solvent. To determine whether occupation of ferroxidase sites and site C is time-dependent, apo-PmFTN crystals were soaked for 5 min, 45 min, 4 h, and overnight in 2 mm ferrous sulfate. Structures of crystals exposed to ferrous iron for 4 h or longer revealed iron bound in all three sites as well as up to two additional sites at the inner surface of the ferritin sphere (Figs. 2*B* and 3*A*). The latter two sites may serve as the nucleation site for formation of the mineral core. The iron in site A is coordinated by the conserved residues Glu-15, Glu-48, and His-51. Coordination of the iron bound in site B is by 2–3 glutamate residues, depending on the subunit. A bridging oxygen atom is modeled between sites A and B and between sites B and C (Fig. 2*B*). A second solvent oxygen atom is coordinated to iron in site A. The bridging solvent molecule between sites A and B might be a bridging oxo group rather than a water molecule, building a diferric-oxo bridge as seen in other ferritins (9). Sample electron density for the overnight-soaked structure at the ferroxidase site including the bridging ligand is presented in Fig. 2*D*. In all subunits, the side chain carboxylate of Glu-48 is a bridging ligand to sites A and B, whereas Glu-94 coordinates to iron bound to site B only. Glu-130 adopts multiple conformations depending on the soaking time and is observed coordinated to iron atoms in site B and/or site C. In the overnight-soaked structure, Glu-130 is tilted toward the iron in site B, whereas in the 4-h-soaked structure, it is predominately coordinated to site C (Fig. 3, *A* and *B*). In addition to Glu-130, the site C iron is coordinated by Glu-47 and Glu-44.

Iron occupancy of the sites A, B, and C varies depending on the incubation time of the crystals in ferrous iron in the presence of dioxygen (Table 2). Iron is observed in site A only after soaking for 45 min (Fe (45)), whereas after 5 min (Fe (5)), iron partially occupies site B in six of eight crystallographic subunits. In the other two subunits, a water molecule is modeled at the equivalent position. The distance between the iron atoms in sites A and B is ∼3.8 Å in Fe (45), which decreases to ∼3.6 Å upon overnight soak. In general, site C, which is situated between the ferroxidase site (sites A and B) and the mineral core, has the lowest overall iron occupancy (40–60%). Only in crystals incubated in ferrous iron for 4 h and longer was iron bound to site C. In the subunits with water bound to site C, the side chain of Glu-130 adopts an alternative rotamer such that it is directed away from the site and toward the core.

**TABLE 2:** **Range of iron and zinc ion occupancy observed in ferroxidase sites A, B, and C of PmFTN aerobically soaked crystals** Soaking time for each metal is indicated in parentheses in minutes unless otherwise indicated. o.n., overnight.

	Fe (5)	Fe (45)	Fe (4 h)	Fe (o.n.)	Zn (1 h)
Site A	H_2_O	Fe (75–90%)	Fe (75–90%)	Fe (85–95%)	Zn (100%)
Site B	Fe (35–50%)	Fe (40–50%)	Fe (75–80%)	Fe (80–85%)	Zn (85–90%)
Site C		H_2_O	Fe (50–60%)	Fe (40–50%)	Zn (60–75%)
Core metal atoms			Fe (50–60%)	Fe (30–35%)	
			Fe (30–50%)		

##### PmFTN Binds Zn(II)

Zinc ions are competitive inhibitors of ferritin and bacterioferritin (14). Crystal structures of Zn(II)-inhibited ferritin confirm that this metal binds to the ferroxidase site and site C. The zinc ions are coordinated by the same residues as seen in the structures from aerobically iron-soaked crystals (Fig. 2*C*). Glu-130 is predominately coordinated to the zinc ion in site C, although in three of the monomers of the asymmetric unit, Glu-130 adopts two equal occupancy conformations, one that coordinates to the zinc ion in site B, and the other that coordinates to the zinc ion in site C (Fig. 3*C*). Furthermore, the zinc ion at site A is modeled as coordinated by two water molecules, one of which is bridging to site B. This arrangement of coordinated solvent is similar to that observed in the iron-soaked structures. In the case of iron, the modeled oxygen atom may be an oxo bridge rather than water. An overlay of the zinc structure with the iron structures showed that the zinc atom in site B is slightly shifted toward site A and that the zinc atom in site C is closer toward the core as compared with the iron.

##### Stopped-flow Absorbance Spectroscopy

**FIGURE 4.**
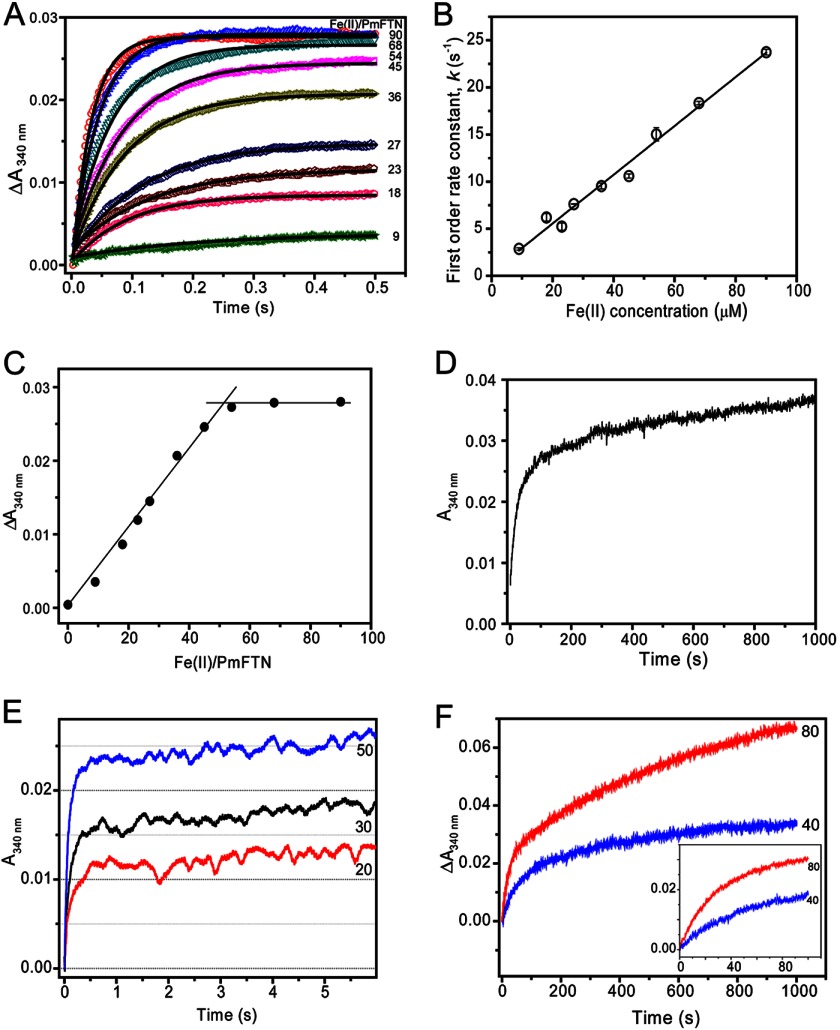
**Kinetic analyses of Fe(II) oxidation catalyzed by PmFTN.** *A*, stopped-flow measurements of Δ*A*_340 nm_ as a function of time following additions of variable Fe(II) (as indicated) to apo-PmFTN. Each of the *A*_340 nm_ traces were fit to a single exponential function (*solid lines*). *B*, plot of observed (pseudo-first order) rate constants obtained from fitting the data in *A* as a function of Fe(II) concentration. Standard errors (*error bars*) are shown, and a linear fit of the data is drawn. *C*, plot of total amplitude of absorbance changes at 340 nm at 0.5 s (data from *A*) as a function of Fe(II) added per PmFTN protein. The two clear phases are highlighted, intersecting at ∼50 Fe(II) ions per protein. *D*, stopped-flow *A*_340 nm_ measurements as a function of time following addition of 100 Fe(II) ions per apo-PmFTN. The initial very rapid oxidation of 48 Fe(II) ions per protein is not captured well on this extended time scale, but the subsequent, much slower oxidation of Fe(II) in excess of that needed to saturate the ferroxidase center sites is observed clearly. *E*, stopped-flow measurement of *A*_340 nm_ over an extended time period revealing small increases in absorbance following the rapid oxidation phase. The number of Fe(II) ions per apo-PmFTN is indicated. *F*, stopped-flow measurements of absorbance changes at 340 nm following the addition of 40 or 80 Fe(II) ions per protein to a sample of PmFTN previously treated with 48 Fe(II) ions per protein under aerobic conditions. The incubation time between Fe(II) additions was 30 min. For all experiments, PmFTN (1 μm) was in 0.1 m MES, pH 6.5.

Stopped-flow experiments were performed to monitor kinetics of iron oxidation after ferrous iron addition to apo-PmFTN. Absorption was measured at 340 nm, which reports on the oxidation of Fe(II) to Fe(III). Fig. 4*A* shows the absorption changes following the mixing of 1 μm apo-PmFTN with a variable amount of ferrous iron as a function of time. Fe(II) oxidation occurred extremely rapidly with completion for all additions within 0.5 s. For the addition of ∼50 Fe(II) ions per protein (two Fe(II) ions per subunit), the *t*½ was <50 ms, demonstrating a rate of reaction that is an order of magnitude faster than those measured for EcFtnA and human H ferritin under comparable conditions (15, 16). Each trace fitted well to a single exponential function (Fig. 4*A*), giving an observed (pseudo-first order) rate constant, *k*_0_, for each addition. Fig. 4*B* shows a plot of *k*_0_ as a function of Fe(II) concentration. Remarkably, there is a linear relationship, demonstrating a first order dependence of the rate of oxidation on the concentration of Fe(II). This observation is significant because it indicates that Fe(II) binding, rather than Fe(II) oxidation, is the rate-determining step of the reaction, consistent with the unprecedented overall rate of Fe(II) oxidation. The slope of the line gives an apparent second order rate constant, *k*, of 2.54 ± 0.10 × 10^5^ m^−1^ s^−1^.

A plot of Δ*A*_340 nm_ at 0.5 s *versus* Fe(II) added per PmFTN complex (Fig. 4*C*) reveals that the rapid oxidation phase is saturated at ∼50 iron ions per protein or two per subunit. Such behavior is characteristic of ferritins in which the ferroxidase center is the initial site of rapid Fe(II) oxidation. Clearly, Fe(II) added in excess of that required to saturate the ferroxidase center is not oxidized at a rate close to that of the initial oxidation phase, and so we conclude that Fe(II) does not undergo rapid oxidation at the third site (site C). This is demonstrated in Fig. 4*D*, which shows changes in *A*_340 nm_ over 1000 s following the addition of 100 Fe(II) ions per protein. Although the first ∼50 Fe(II) ions are oxidized extremely rapidly, the subsequent 50 are not; in fact, oxidation is not complete at 1000 s. Thus, the kinetic distinction between these phases is unusually clear.

The partial occupancy of iron in the ferroxidase sites A and B after prolonged exposure to Fe(II) under aerobic conditions (Table 2) indicates that iron is likely to be mobile following oxidation at the ferroxidase center. Thus, *A*_340 nm_ was monitored over a longer time period (Fig. 4*E*). The data show that a small increase in absorption occurs during the few seconds following the rapid oxidation phase. This increase was observed at all levels of Fe(II) additions, including those that are substoichiometric, and so these changes are not due to oxidation of Fe(II) bound elsewhere. Thus, these absorption changes may be attributed to reorganization of iron in the ferroxidase center and possibly site C following the initial rapid oxidation.

Mobility of iron following oxidation of Fe(II) at the ferroxidase center could result in complete loss of Fe(III) from the center, leading to regeneration of the apo form. This form would be expected to exhibit the rapid oxidation phase observed above (Fig. 4*A*). To determine whether the rapid oxidation phase is recovered, PmFTN was loaded with 48 Fe(II) ions per 24-mer and then subsequently incubated for 30 min or 20 h. Then, a further aliquot of Fe(II) (either 40 or 80 Fe(II) ions per protein) was added, and changes in *A*_340 nm_ were measured. The data (for 30 min; Fig. 4*F*) show that no rapid phase of Fe(II) oxidation is observed. Two distinct phases are observed, although it is not yet clear what these correspond to. Data for 20 h are similar (not shown). Clearly, there is no full regeneration of the apo form of PmFTN following oxidation of the initial addition of Fe(II).

## DISCUSSION

Iron storage ferritins are the archetype of the ferritin-like superfamily of proteins, which includes the enzymes ribonucleotide reductase and methane monooxygenase. A common feature is a catalytic di-iron site where dioxygen is reduced. In ferritin, Fe(II) is the source of electrons, and the resulting oxidized Fe(III) is deposited in the mineral core. A key question in ferritin function is the mechanism of iron mobilization in and out of the ferroxidase site and the core. Iron was visualized in the ferroxidase site (sites A and B) and a third site C of PmFTN. The residues interacting with the iron in site A (Glu-15, Glu-48, and His-51) are conserved with those of all characterized eukaryotic and prokaryotic ferritins. In addition to Glu-48, Glu-94 coordinates iron in site B and is also conserved.

A third iron site is generally not associated with ferritin ferroxidase sites from vertebrates; however, a site C has been observed in some non-heme ferritins from bacteria and archaea, for example in *E. coli* (EcFtnA) (16, 17), *Pyrococcus furiosus* (18), and *Archaeoglobus fulgidus* (19). Nevertheless, these sites differ from that of PmFTN in terms of the number and origin of glutamate residues. Of these residues, Glu-130 is in common, is observed to bridge the iron atom of sites B and C (Fig. 3), and is a conserved residue in prokaryotic ferritins. In the PmFTN structure soaked aerobically for more than 4 h, Glu-130 is a ligand to iron atoms in sites B and C. In contrast, in the earlier PmFTN structure soaked in iron for 10 min by Marchetti *et al.* (6), the iron in site C is coordinated by only Glu-44. In all known prokaryotic ferritins, Glu-44 is substituted by histidine. The equivalent His-46 in EcFtnA is proposed to orient Glu-130 so it can bind iron ions in sites B and C as well as gating the passage of the metal through these sites (17). Glu-44 and Glu-130 in PmFTN may have a similar function of gating the passage of iron from site B to site C.

As the aerobic iron soaking time increases from minutes to hours, iron is observed first in site B followed by site A and eventually occupies all three sites (Table 2). In contrast, only site A is occupied by Fe(II) in crystals of PmFTN under anaerobic conditions even though the crystals were exposed to 2 mm Fe(II) for over an hour (Fig. 2*A*). This observation is in contrast to short (∼1-min) aerobic Fe(II) soaking experiments with frog ferritin in which both sites A and B are occupied (9). Interestingly, the interiron distance in some subunits of frog ferritin is comparable with that observed with Cu(II) as a proxy for Fe(II) (∼4.3 Å). Longer exposure to ferrous iron results in a shortening of the di-iron interatomic iron distance to ∼3.1 Å. In PmFTN, iron occupancy at both sites is only observed after prolonged iron exposure, and the site A and B intermetal distance of less than 3.8 Å decreases only slightly with time to 3.6 Å, suggesting that in the structures where iron is bound to both sites it is in the Fe(III) state. Note that with the data presented here we are not able to directly determine the oxidation state of iron bound to the crystals under aerobic conditions, including that in site B in Fe (5). A single high affinity and two low affinity Fe(II) binding sites were identified in *P. furiosus* ferritin by calorimetry in the absence of dioxygen (20). Site-directed mutagenesis was used to propose assignment of the high affinity site to site A, consistent with our anaerobic crystallographic observations in PmFTN.

Kinetic measurements of PmFTN iron oxidation revealed an extremely rapid initial oxidation phase involving the binding and oxidation of two ferrous iron. The first order dependence of the rate of ferroxidase center oxidation on the concentration of Fe(II) demonstrates a close link between binding and oxidation events such that they cannot be distinguished. Thus, oxidation occurs immediately upon Fe(II) binding to PmFTN, and the binding event can be viewed as the slow step of the reaction. This is in contrast to previous reports of ferritins in which binding and oxidation are considered to be kinetically distinct events. Measurement of Fe(II) binding kinetics is not generally straightforward, although it was possible for EcBFR because Fe(II) binding caused a perturbation of absorbance due to the heme groups. In that case, Fe(II) binding occurred on a much shorter time scale than the subsequent Fe(II) oxidation. Interestingly, Fe(II) binding to EcBFR occurred with a second order rate constant of 2.5 × 10^5^ m^−1^ s^−1^ (at 30 °C) (15), a value similar to that measured here (at 25 °C) for PmFTN-catalyzed Fe(II) oxidation.

An oximetric assay previously showed that the ferroxidase reaction of PmFTN is associated with consumption of dioxygen in a ratio of 1.9 ± 0.2 Fe(II):O_2_ (6). Furthermore, addition of catalase to the assay solution resulted in the stoichiometric regeneration of O_2_, indicating production of H_2_O_2_ by the ferroxidase reaction as seen with ferritins (21) but not bacterioferritins (14) or Dps ferritins (22). In contrast, the oxidation stoichiometry of EcFtnA is 3–4 Fe(II) ions per O_2_, which is suggested to be a consequence of the binding of three iron atoms (one at site C) and a possible fourth iron at an unknown metal site, leading to reduction of O_2_ to water rather than hydrogen peroxide (16). The ferroxidase reaction in EcFtnA is similar to that observed in human H chain ferritin, although it is more complex due to the third iron in site C (17). Site C, however, is not essential for ferroxidase activity in EcFtnA as site C variants showed only a slight decrease in the overall oxidation rate but the expected stoichiometry of two ferrous iron per dioxygen (16). In contrast, although a third iron site is present in PmFTN, a 2:1 Fe:O_2_ stoichiometry is retained. The kinetic data reported here support the conclusion that only two Fe(II) ions are initially oxidized per subunit. A key structural difference is that site C in PmFTN is only 3.5–3.7 Å from site B, whereas site C in EcFtnA is 7–8 Å from the A/B pair. Third iron binding sites were observed in EcBFR as well as human mitochondrial ferritin (23, 24). However, these iron sites were observed to be at the core surface and are more likely involved in the nucleation/mineralization process rather than in ferroxidase center-catalyzed iron oxidation (25).

Only ferroxidase site A is occupied by ferrous iron in the anaerobic crystal; however, stopped-flow data show saturation of the rapid phase 2 after binding of 2 ferrous iron eq per monomer of PmFTN. Together these results point to stepwise binding of the ferrous iron and dioxygen to the ferroxidase site. A model can be proposed in which one ferrous iron binds to site A followed by the binding of the oxidant. Only when the latter is bound can the second ferrous iron bind to site B. Thus, at the moderate iron concentrations (2 mm) used for soaking experiments, a second Fe(II) ion is not observed at the center in the absence of the oxidant (dioxygen). We note that a similar model was proposed for the two Dps proteins from *Bacillus anthracis* (26). These are 12-mer (mini)ferritins that contain intersubunit dinuclear ferroxidase centers that are distinct from those of the 24-mer (maxi)ferritins but nevertheless share some common features. For PmFTN, such a model accounts for the observed rate dependence on Fe(II) because once the second Fe(II) binds oxidation can proceed immediately. Thus, ferrous iron binding to site B of the ferroxidase site is proposed to be the rate-determining step.

Evolution of a rapid dioxygen-driven reaction is of value to an organism living in an iron -limited environment. Under low oxygen conditions, a PmFTN 24-mer would sequester at most 24 ferrous ions, making more iron available for metabolic processes. In the presence of dioxygen and an environmental iron pulse, pre-existing PmFTN would rapidly store newly accumulated iron, preventing the generation of reactive oxygen species and providing a buffer for induction of PmFTN expression.

Zn(II) is an inhibitor of EcFtnA and is proposed to compete with ferrous iron for the dinuclear center and consequently inhibit oxidation at these sites (27). In the crystal structure, Zn(II) does bind to sites A and B but is not observed in site C (17). Nonetheless, from the Fe:O_2_ stoichiometry of the EcFtnA reaction, all three sites were proposed to bind Fe(II) during catalysis. Crystal structures of Zn(II) complexes of EcBFR and human mitochondrial ferritin have Zn(II) bound at sites A and B of the ferroxidase center, consistent with a proposed model of Fe(II) binding (24, 28). However, anaerobic Fe(II) complexes for these systems are not available in the literature. We have directly compared Zn(II) and Fe(II) binding in PmFTN (Fig. 2). Zn(II) bound to sites A, B, and C in contrast to the two sites observed in EcFtnA. Furthermore, overall Zn(II) occupancy of the three metal sites resembles iron bound in the presence of dioxygen in PmFTN rather than mimicking Fe(II) binding. Thus, the use of Zn(II) and likely other metal ions as analogs of Fe(II)/Fe(III) may not identify the correct binding sites in other ferritins.

Two models have been proposed for the mechanism of ferroxidation by ferritins and bacterioferritins. In one model, the ferroxidase site functions as a substrate site as seen in human H ferritin (29), EcFtnA (30), and frog M ferritin (31). Ferrous iron binds to the ferroxidase site, and after oxidation, ferric iron rapidly migrates to the mineral core. In a second model, first described for EcBFR (23) and *P. furiosus* ferritin (32), the ferroxidase site is a stable di-iron site that functions as a cofactor after the binding of 2 eq of ferrous iron per subunit. Additional ferrous ions are then added directly to the mineral core, and the ferroxidase site functions solely in oxygen or peroxide reduction. Recently, Honarmand *et al.* (20) proposed a unifying mechanism in which the Fe(III) product at the ferroxidase site remains bound to the ferroxidase site but is rapidly displaced by incoming Fe(II). A prediction of this revised model is the observation of a fully Fe(III)-loaded ferroxidase site in crystals after prolonged soaking in Fe(II). The ferroxidase site of PmFTN was not fully occupied after soaking aerobic crystals in ferrous iron for 45 min, suggesting that iron movement occurred at the ferroxidase site during iron loading with 2 mm ferrous iron over an extended time period. Small absorbance changes immediately following oxidation of Fe(II) at the ferroxidase center are consistent with this conclusion. Nevertheless, for PmFTN, the rapid oxidation of ferrous iron was not regenerated upon up to 20 h incubation, indicating that iron remains present at least in part at the ferroxidase site. Thus, if Fe(III) is displaced, the subsequent iron oxidation is much slower than the initial oxidation. Thus, the mechanism of mineralization in PmFTN appears to be more complex with partial iron migration to the core.

Our data indicate that site A of the ferroxidase center has a higher affinity than site B for Fe(II) under anaerobic conditions. The two distinct kinetic phases observed after the second addition of 48 Fe(II) ions may also be related to slow iron migration to the core likely involving the third iron binding site (site C) and perhaps other sites along a path to the cavity. In frog ferritin, the transit of Fe(III) from the ferroxidase center to the cavity has been shown to occur via a pathway through the subunit toward a 4-fold channel (33). Site C may function to direct Fe(III) along a different path to the mineral core. In EcBFR, the two ferroxidase center sites were fully occupied after 2.5 min of soaking, and occupancy was not affected following oxidation, suggesting that a distinct mechanism is in operation (23).

## Footnotes

2 The abbreviations used are:

H: heavy
PmFTN: *P. multiseries* ferritin
EcBFR: *E. coli* bacterioferritin
EcFtnA: *E. coli* ferritin
TCEP: tris(2-carboxyethyl)phosphine
Fe (5): Fe (45), Fe (75), Fe (2 h), Fe (4 h), and Fe (o.n.) refer to the structures of PmFTN from crystals soaked in solutions of ferrous iron for 5 min, 45 min, 75 min, 2 h, 4 h, and overnight, respectively
Zn (1 h): refers to the structure of PmFTN from crystals soaked in Zn(II) for 1 h.
